# Supplementary material for: Outcomes of prognostication in people living with advanced cancer: A qualitative study to inform a Core Outcome Set
Source: PLoS One. 2024 Jul 11;19(7):e0306717. doi: 10.1371/journal.pone.0306717 (PMC11239020; doi:10.1371/journal.pone.0306717)
Supplement: S2 File — (DOCX) [file pone.0306717.s002.docx]

**CAREGIVER INTERVIEW GUIDE**

INTERVIEWS

**Introduction**

Thank you for agreeing to participate in this interview. We are interviewing you to explore the perceptions and experiences of individuals regarding the outcomes of prognostication in palliative care. As you probably know, prognostication is defined as the process of making predictions about the future, such as the likely outcome or course of a disease, the chance of recovery or recurrence. In particular, we are interested in looking at the outcomes of providing prognostic information in palliative care. As you've seen in the information sheet, this interview is part of a wider study, looking to develop a Core Outcome Set to assess the impact of end-of-life prognostication in palliative cancer care. A Core outcome set is a set of main outcomes that can be measured and reported in future clinical studies, so that those studies can be compared and combined.

There are no right or wrong answers to any of the questions, I’m interested in your own experiences and opinions. Participating in this study is voluntary and your decision to participate, or not participate, will not affect your rights or the care your [mother/father/spouse/friend, etc.]* currently receives.

The interview should take approximately 30 minutes to one hour, depending on how much information you would like to share. With your permission, I would like to audio record the interview because I don't want to miss any of your comments. All responses will be kept confidential. This means that your interview responses will only be shared with the research team, and we will ensure that any information we include in our report does not identify you as the respondent. You may say you don’t want to answer any question or stop the interview at any time and for any reason. Do you have any questions about what I have just said?

*If YES, answer questions and ask if the participant is happy to proceed to the interview.*

*If NO, proceed*

May I turn on the digital recorder?

*personalise and use throughout interview. Be as conversational as possible, say "your mum" or "your dad" or the care recipient's name/pseudonym if that is the term the participant uses to describe their relative/care recipient.

__________________________________________________________________________

*Please note that this guide only represents the main themes to be discussed with the participants and as such does not include the various prompts that may also be used (examples given for each question). Non-leading and general prompts will also be used (please refer to last page).*

**Establishing Rapport**

Before we begin, it would be helpful if you could tell me a little bit about why you were interested in taking part in this study.

**Semi-structured interview guide**

1. Were you present when [care recipient] was told their prognosis?

**Prompts:**

- *If YES:* By whom?
- *If NO:* When did you first hear about [care recipient's] prognosis? Who told you? When?

1. When did this happen? How long ago?

**Prompts:**

- If you cannot remember specific date, perhaps just the year?

1. Would you rather have been told the prognosis sooner or later than actually happened?

**Prompts:**

- And why is that?

1. Did you find it helpful or unhelpful knowing [care recipient’s] likely prognosis?

**Prompts:**

- In what ways?
- How did knowing their prognosis affect your feelings
- How did knowing their prognosis affect your relationship with them?
- How did knowing their prognosis affect how you made plans?
- How did knowing their prognosis affect you financially?

1. Do you think this would have affected any aspect of [care recipient’s] care?

**Prompts:**

- What? How?
- How did knowing their prognosis affect choices and decisions made about their care?

What kind of effect on your lives do you think would it have had if you had known sooner/later?

**Prompts:**

How? Why?

1. Is there anything you would have done differently if you didn’t know their prognosis?

**Prompts:**

- What? Why?

1. What information do you think is important for a palliative care clinician to tell patients and caregivers about their prognosis?

**Prompts:**

- There are different ways that people can be told about the likely prognosis. Some doctors use very broad general terms, like good or not so good; others talk about probabilities, like 40% chance. Some are a bit more specific, like days or weeks, or some will be as specific as they can be, and tell their patients the length of time they expect, like three months or five or six weeks. What do you think about these approaches? Do you think any of them are better or worse?
- Are there any other details that matter?
- Why are these things important?

1. How do you think patients and their caregivers should be told about the likely prognosis?

**Prompts:**

- How? Why?
- Would you like information to be available online, or only provided in person by a professional?
- Who should start talking about prognosis? The patient or the doctor or nurse?

1. Which of the things (or *effects*) that you have mentioned do you consider the most important?

**Prompts:**

- Which has affected you the most?
- Why is/was this important?

**Conclusion**

1. This is my last question. Are there any other good or bad things about knowing [care recipient’s] prognosis that you can think of that we haven't discussed today?

_________________________________________________________________________

**End**

Thank you very much for your time and the information you shared today.

Would you like me to let anyone looking after you or [care recipient] know about anything you have told me about their care?

Would you like to be kept informed of the results of my study? If so, are you happy for me to keep your contact details?

Non-leading and general prompts:

- Can you tell me more about that?
- Tell me what that was like for you?
- Why is that?
- Can you clarify more about [insert]?
- You mentioned [insert], can you describe what you mean by that?
- Reflect on answer, summarise to check, and prompt
