# Supplementary material for: Outcomes of prognostication in people living with advanced cancer: A qualitative study to inform a Core Outcome Set
Source: PLoS One. 2024 Jul 11;19(7):e0306717. doi: 10.1371/journal.pone.0306717 (PMC11239020; doi:10.1371/journal.pone.0306717)
Supplement: S3 File — (DOCX) [file pone.0306717.s003.docx]

**CLINICIAN INTERVIEW GUIDE**

INTERVIEWS

**Introduction**

Thank you for agreeing to participate in this interview. We are interviewing you to explore the perceptions and experiences of individuals regarding the outcomes of prognostication in palliative care. As you probably know, prognostication is defined as the process of making predictions about the future, such as the likely outcome or course of a disease, the chance of recovery or recurrence. In particular, we are interested in looking at the outcomes of providing prognostic information in palliative care. As you've seen in the information sheet, this interview is part of a wider study, looking to develop a Core Outcome Set to assess the impact of end-of-life prognostication in palliative cancer care. A Core outcome set is a set of main outcomes that can be measured and reported in future clinical studies, so that those studies can be compared and combined.

There are no right or wrong answers to any of our questions, we are interested in your own experiences and opinions. Participating in this study is voluntary and your decision to participate, or not participate, will not affect your rights or your career.

I expect this interview to take between 30 minutes and an hour, depending on how much information you would like to share. With your permission, I would like to audio record the interview because I don't want to miss any of your comments. All responses will be kept confidential. This means that your interview responses will only be shared with the research team, and we will ensure that any information we include in our report does not identify you as the respondent. You may decline to answer any question or stop the interview at any time and for any reason. Do you have any questions about what I have just explained?

*If YES, answer questions and ask if the participant is happy to proceed to the interview.*

*If NO, proceed*

May I turn on the digital recorder?

__________________________________________________________________________

*Please note that this guide only represents the main themes to be discussed with the participants and as such does not include the various prompts that may also be used (examples given for each question). Non-leading and general prompts will also be used (please refer to last page).*

**Establishing Rapport**

Before we begin, it would be helpful if you could tell me a little bit about why you were interested in taking part in this study.

**Semi-structured interview guide**

1. How do you usually approach prognostic conversations?

**Prompts:**

- Do you use a specific algorithm/method to deliver a prognosis?
- What information do you think patients and their caregivers need to know about their prognosis?
- Do you use any resources to assist them in understanding their prognosis? For example, pamphlets, pictures, etc.? If not, why not? If so, how useful, or not useful do you think they were in helping them to understand their prognosis?
- Do you think it’s better to provide estimates in terms of days, weeks, or months, or as probabilities, or using general broad terms such as good or not so good?
- Do you refer patients and their caregivers to further support? What/who?

1. In your experience, do you think that providing a prognosis is helpful or unhelpful to patients and their caregivers?

**Prompts:**

- In what ways?
- How do you think knowing their prognosis might affect how they make plans?
- How do you think knowing their prognosis might affect them financially?

1. In your experience, do you think that providing a prognosis is helpful or unhelpful clinically?

**Prompts:**

- In what ways?
- For example, how do you think knowing a patient's prognosis might help with planning care?
- What about with prioritising caseload?
- What about admission and discharge decisions?
- What about communication with colleagues?
- What about referring patients to hospice services?

1. You probably find that patients and their families or friends are upset after [you/their clinician] have shared their prognosis with them. Do you notice any other changes in patients?

**Prompts:**

- For example, does their physical health improve, decline, or stay the same? Can you provide an example of that?
- What about their mental health?
- What about their relationships?
- What do you think might be the reasons for these changes?

1. Do you think providing a prognosis might affect any particular aspect of a patient’s care?

**Prompts:**

- What? How?

1. Do you measure any outcomes or effects of providing prognosis to patients and their caregivers?

**Prompts:**

- If yes, how?
- If no, do you have any thoughts on how we might measure any effects or outcomes of providing prognoses?

1. Which of the things (or *effects*) that you have mentioned do you consider the most important?

**Prompts:**

- Which do you think would affect patients and/or caregivers the most?
- Why is/was this important?

**Conclusion**

1. This is my last question. Are there any other good or bad things about providing a prognosis that you can think of that we haven't discussed today?

_________________________________________________________________________

**End**

Thank you very much for your time and the information you shared today.

Would you like to be kept informed of the results of my study? If so, are you happy for me to keep your contact details?

Non-leading and general prompts:

- Can you tell me more about that?
- Tell me what that was like for you?
- Why is that?
- Can you clarify more about [insert]?
- You mentioned [insert], can you describe what you mean by that?
- Reflect on answer, summarise to check, and prompt
